# HBO1 induces histone acetylation and is important for non-small cell lung cancer cell growth

**DOI:** 10.7150/ijbs.72526

**Published:** 2022-05-09

**Authors:** Teng-fei Chen, Hui-fei Hao, Yan Zhang, Xiao-yu Chen, Hua-si Zhao, Rui Yang, Ping Li, Ling-xiao Qiu, Yong-hua Sang, Chun Xu, Shao-xia Liu

**Affiliations:** 1 Respiratory Department I, The First Affiliated Hospital of Zhengzhou University, Zhengzhou, China; 2 Key Laboratory of Neuroregeneration of Jiangsu and Ministry of Education, Nantong University, Nantong, China; 3 Department of Radiotherapy and Oncology, Affiliated Kunshan Hospital of Jiangsu University, Kunshan, China; 4 Changshu Hospital Affiliated to Nanjing University of Chinese Medicine, Changshu, China; 5 Department of Cardiothoracic Surgery, the Second Affiliated Hospital of Soochow University, Suzhou, China; 6 Department of Thoracic Surgery, Institute of Thoracic Surgery, The First Affiliated Hospital of Soochow University, Suzhou, China

## Abstract

We examined the expression and the potential biological function of HBO1 in non-small cell lung cancer (NSCLC). TCGA and Oncomine databases showed that *HBO1* transcripts were elevated in NSCLC. Furthermore, in local NSCLC tumor tissues HBO1 expression was higher than that in matched adjacent lung tissues. In primary and immortalized NSCLC cells, HBO1 shRNA robustly inhibited cell viability, proliferation and migration. Moreover, HBO1 knockout by CRISPR/Cas9 induced significant anti-tumor activity in NSCLC cells. Conversely, ectopic HBO1 overexpression in primary NSCLC cells increased proliferation and migration. H3-H4 histone acetylation and expression of several potential oncogenic genes (*CCR2*, *MYLK*, *VEGFR2* and *OCIAD2*) were significantly decreased in NSCLC cells with HBO1 silencing or knockout. They were however increased after HBO1 overexpression. Intratumoral injection of HBO1 shRNA-expressing adeno-associated virus hindered the growth of A549 cell xenografts and primary NSCLC cell xenografts in nude mice. H3-H4 histone acetylation as well as expression of HBO1 and HBO1-dependent genes were decreased in HBO1-silenced NSCLC xenograft tissues. An HBO1 inhibitor WM-3835 potently inhibited NSCLC cell growth. Together, HBO1 overexpression promotes NSCLC cell growth.

## Introduction

It is important to explore novel molecular targets essential for uncontrolled non-small cell lung cancer (NSCLC) cell growth 1-5. Dysregulation in epigenetics is closely associated with pathogenesis and progression of NSCLC 6, 7. Histone acetylation is a key epigenetic mechanism of gene expression 8-10. Histone deacetylase (HDAC) and histone acetyltransferase (HAT) and are two primary families of enzymes responsible for histone acetylation 10, 11. Sun *et al.,* showed that nuclear glycogen metabolism could provide substrates for histone acetylation to increase NSCLC cell proliferation 12. Mi *et al.,* reported that YEATS domain-containing 2 (YEATS2) acted as a reader of histone H3 acetylation, required for transcriptional activation of multiple key genes for NSCLC tumorigenesis 13.

One HAT family protein, HBO1, also known as KAT7 or MYST2, catalyzes H4 and H3 histone acetylation at multiple lysine residues 14. HBO1 is required for acetyl-CoA binding and histone acetylation 15. Through association with native subunits and cofactors, HBO1 regulates multiple key physiological functions and behaviors. It is important for gene transcription and expression, cell self-renewal, immune-response regulation and development 15-20. HBO1 association with BRPF scaffold proteins is vital for histone H3K14 acetylation (H3K14ac) 15. Moreover, HBO1 association with JADE (JADE1/2/3) can catalyze histone H4 acetylation 16, 17, 21. HBO1-mediated H3K14ac is required for replication origin activation. HBO1-catalized histone H4 acetylation is shown to promote DNA replication licensing 16-20.

Zhong* et al.,* demonstrated that HBO1 is overexpressed in hepatocellular carcinoma (HCC), and it is required for cancer growth. Conversely, HBO1 silencing by shRNA or CRISPR/Cas9-induced HBO1 knockout (KO) potently inhibited HCC growth 22. Gao *et al.,* have shown that HBO1 is a novel and important oncogenic gene of osteosarcoma. HBO1 silencing or inhibition (by a first-in-class inhibitor WM-3835) robustly inhibited osteosarcoma cell growth 23. The results of this study will show that HBO1 overexpression is important for NSCLC cell growth.

## Materials and methods

**Chemicals and reagents.** WM-3835 was provided by Dr. Cao's group 24. Cell counting kit -8 (CCK-8), EdU (5-Ethynyl-2'-deoxyuridine), DAPI and TUNEL dyes were all from Invitrogen Thermo-Fisher (Beijing, China). Antibodies were described previously 22, 24.

**Cell culture.** Provided by Dr. Shi's group 25, the primary human NSCLC cells, pNSCLC-1/-2/-3, were derived from three different written-informed consent patients 25. Primary NSCLC cells and established cell lines (A549 and H460) were maintained under the described medium 25. The primary epithelial cells-derived from human lung were also from Dr. Shi 25.

**Human tissues.** Thirteen (13) NSCLC patients (all male, 42 to 78-year old, stage-III-IV), administrated at authors' institutions, were enrolled in this study. The written-informed consent was obtained from each patient. Fresh tumor tissues and matched surrounding normal lung tissues were obtained at the time of surgery and were stored in liquid nitrogen. The fresh tumor specimens of six NSCLC patients with poor over survival (less than 50 months, 19-43 months) and another six tumors of NSCLC patients (age, sex and disease-stage matched) with better overall survival (over 50 months) were provided by the First Affiliated Hospital of Soochow University (Suzhou, China). The protocols of using human tissues and cells were approved by Zhengzhou University's Ethics Committee and were in according to Declaration of Helsinki priciples.

**HBO1 silencing or overexpression**. The GV369 lentiviral constructs encoding six different shRNAs targeting human *HBO1* (sh-HBO1-Sq1 to sh-HBO1-Sq6) were provided by Genechem (Shanghai, China). The lentiviral construct encoding the full-length HBO1 cDNA was provided by Dr. Cao 24. The construct, and the lentivirus helper plasmids, were transfected to HEK-293T cells. The achieved lentiviral particles were enriched and transduced to cultured NSCLC cells (maintained under the polybrene containing complete medium). After 48h, puromycin was added to selected stable cells for another 48h.

**HBO1 knockout (KO)**. A LentiCas9-puro construct (Genechem) was transduced to pNSCLC-1 cells and cultured for 72h. Stable cells were formed after selection using puromycin. Cells were then further transfected with Lenti-HBO1-sgRNA-puro construct (from Dr. Cao 24). A set of five different sgRNAs were tested. The transfected cells were further distributed to 96-well plates and were subjected to HBO1 KO screening. Among the five tested sgRNAs, HBO1 sgRNA-2 (“Sg2”) and HBO1 sgRNA-5 (“Sg5”) resulted in complete *HBO1* KO. The single stable HBO1 KO pNSCLC-1 cells were established.

**Other methods**, including Western blotting, qRT-PCR, CCK-8, colony formation, EdU staining, Transwell assays, TUNEL staining and other apoptosis-related assays were described in the previous studies 25, 26. Same set of protein lysates were always run in parallel “sister” gels to test different proteins. Each blotting data was repeated for five times and similar results were obtained in each time. The mRNA primers utilized in this study were provided by Dr. Cao 24. The uncropped blotting images of the study were listed in **Figure S1**.

**Xenograft study.** A549 cells or pNSCLC-1 primary cells (at six million cells of each mouse) were inoculated to the flanks of the nude mice via subcutaneous (*s.c.*) injection. A549 xenografts or pNSCLC-1 xenografts were established within three weeks. Mice were thereafter assigned randomly into two different groups and were intratumorally injected with the applied adeno-associated virus (AAV). Tumor volume was calculated as described 25. All animal studies were approved by the IACUC and Institute Animal Ethics Review Board of Zhengzhou University.

**Statistical analyses.** The *in vitro* experiments of the study were repeated five times, and each time similar results were obtained. Numeric data were always with normal distribution and were presented as mean ± S. D. (standard deviation). Statistical differences of multiple groups were analyzed by ANOVA and Dunnett's test (SPSS 23.0, Chicago, CA). The difference between two specific groups was evaluated by Student's t test. ***P*** < 0.05 stands for the statistically significant difference.

## Results

### HBO1 overexpression in NSCLC

**Figure 1:**
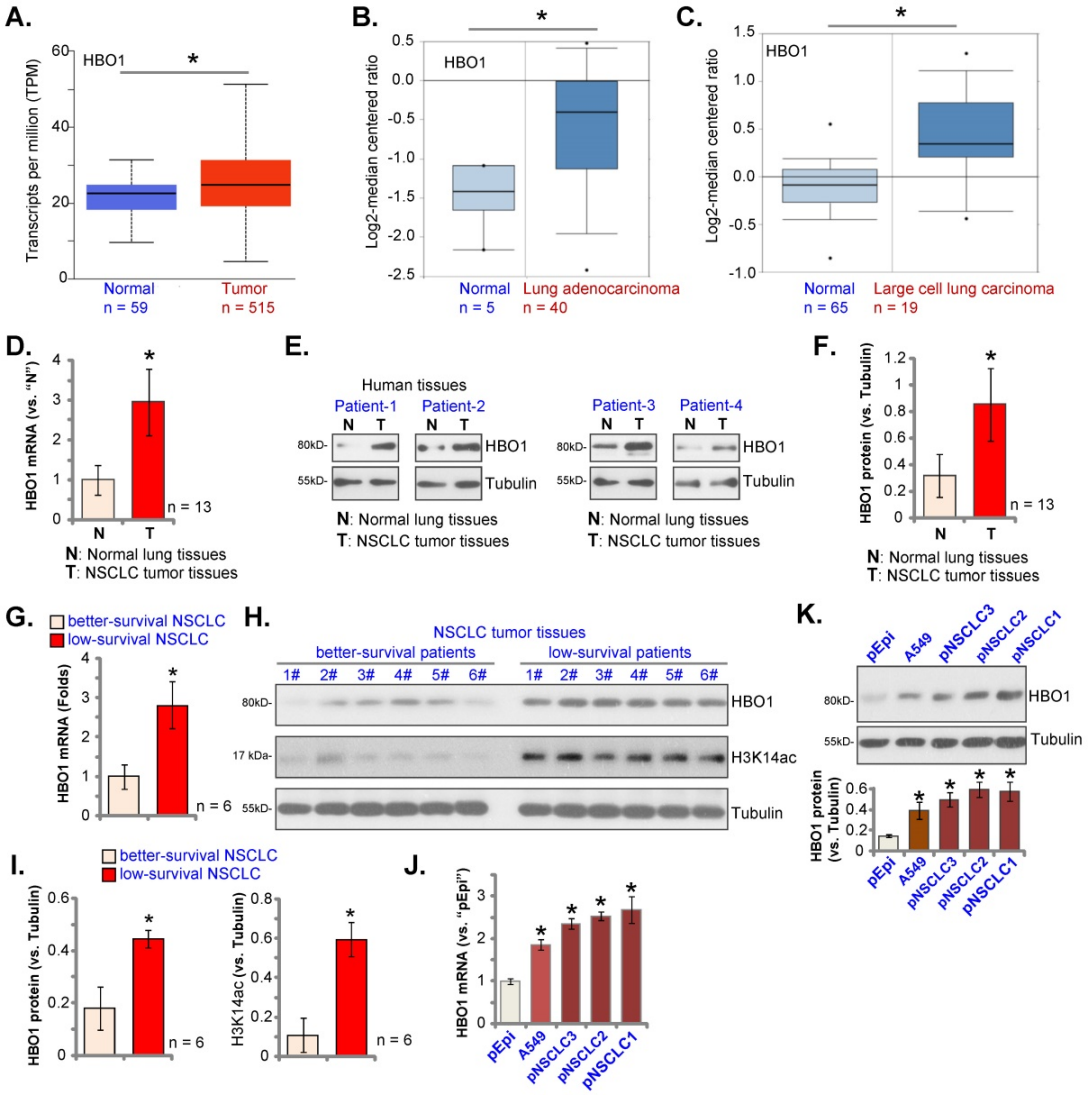
** HBO1 overexpression in NSCLC.** TCGA cohorts show *HBO1* transcripts in 515 cases of lung adenocarcinoma tissues (“Primary Tumor”) and 59 cases of normal lung tissues (“Normal”) (**A**). Oncomine 4.5 database shows the relative *HBO1* mRNA expression in described lung cancer tissues and normal lung tissues (“Normal”) (**B** and **C**). Expression of *HBO1* mRNA and listed proteins in the described NSCLC tumor tissues and normal lung tissues (“N”) as well as in the listed NSCLC cells and normal lung epithelial cells (“pEpi”) were tested by qRT-PCR (**D**, **G** and **J**) and Western blotting (**E**, **F**, **H**, **I** and **K**) assays. *P < 0.05 versus “Normal”/“N” tissues or “pEpi” cells. *P < 0.05 versus “better-survival NSCLC” tissues (**G** and **I**).

TCGA database was first utilized to examine *HBO1* expression in NSCLC tissues. As demonstrated, *HBO1* transcripts in NSCLC tissues (“Tumor”, n = 515) were higher than those in normal lung tissues (“Normal”, n = 59) (Figure **1A**). Oncomine 4.5 database results further showed that *HBO1* mRNA levels in lung adenocarcinoma tissues (n = 40) were significantly higher than those in the normal lung tissues (n = 5, Figure **1B**). Furthermore, *HBO1* mRNA elevation was detected in large cell lung carcinoma (***P*** < 0.05 versus normal lung tissues, Figure **1C**). Therefore, bioinformatics assay results show that *HBO1* is elevated in NSCLC tissues.

HBO1 expression in local human NSCLC tissues was tested next. Thirteen (n = 13) pairs of patient-derived NSCLC tumor tissues (“T”) and matched adjacent normal lung tissues (“N”) were analyzed. *HBO1* mRNA expression in NSCLC tumor tissues was significantly higher than that in normal lung tissues (Figure **1D**). Figure **1E**, showed that HBO1 protein expression was elevated in NSCLC tumor tissues in the four representative patients (“Patient-1” to “Patient-4”). Quantitative analyses integrating all 13 sets of human tissues showed that HBO1 protein upregulation in NSCLC tumor tissues was significant (***P*** < 0.05 versus “N” tissues, Figure **1F**).

To examine whether HBO1 overexpression in NSCLC is associated with patient survival. The tumor specimens of six NSCLC patients with poor over survival (less than 50 months, 19-43 months) and tumors of other six NSCLC patients (age, sex and disease-stage matched) with better overall survival (over 50 months) were obtained. Expression of HBO1 was tested. mRNA and protein expression of HBO1 in tumor tissues of low-survival NSCLC patients was significantly higher than that in tumors of better-survival NSCLC patients (Figure **1G**-**I**). H3K14ac in tumor tissues of low-survival NSCLC patients was also significantly higher than that in better-survival NSCLC patients (Figure **1H** and **I**).

The expression of HBO1 in NSCLC cells was studied. *HBO1* mRNA and protein (Figure **1J** and **K**) levels were relatively low in primary lung epithelial cells (“pEpi”). While HBO1 overexpression was detected in primary NSCLC cells that were derived from three different patients, “pNSCLC-1/2/3”, as well as in the immortalized A549 cell line (Figure **1J** and **K**). These results confirmed HBO1 overexpression in NSCLC.

### HBO1 silencing causes anti-NSCLC cell activity

**Figure 2:**
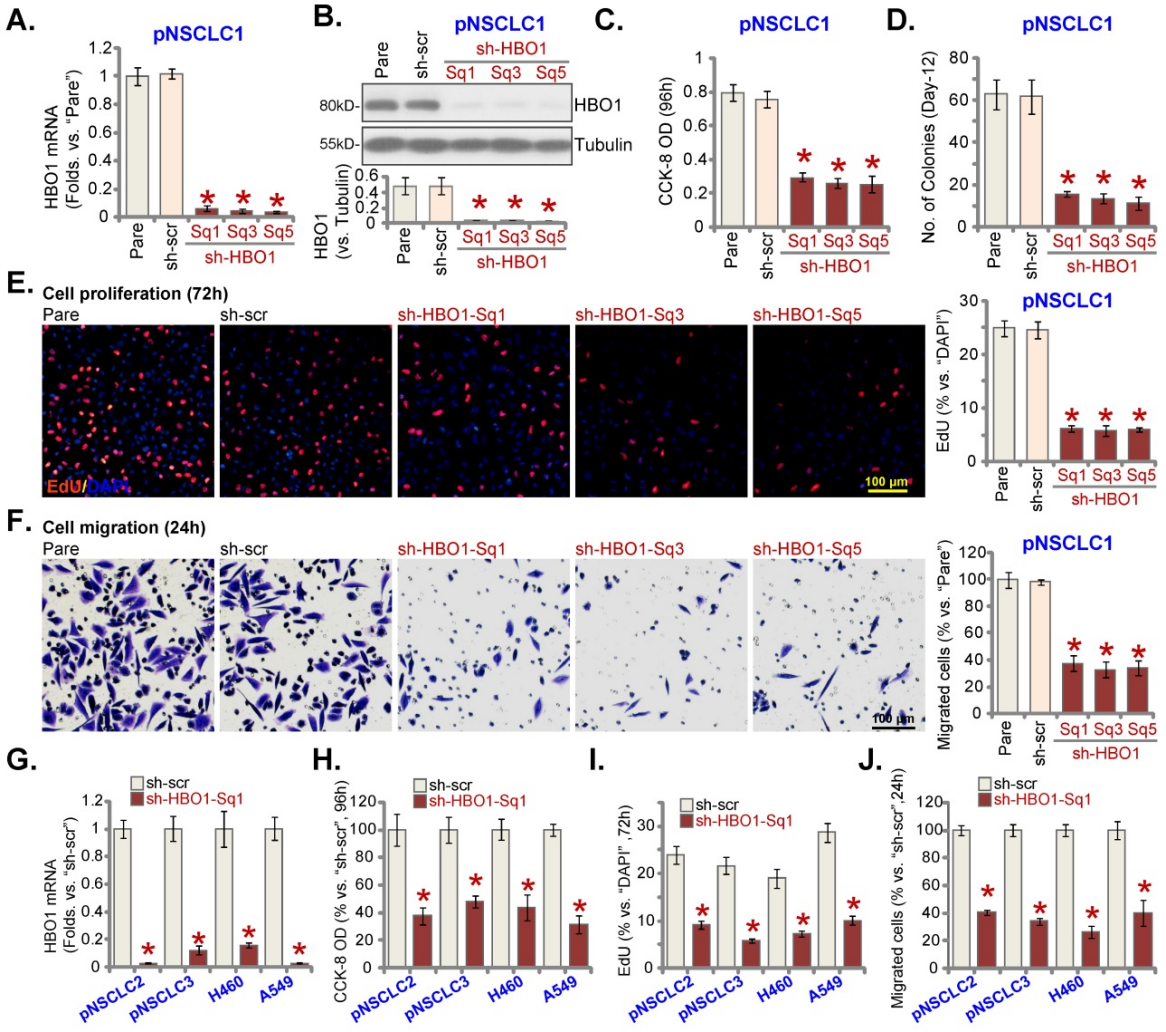
** HBO1 silencing causes anti-NSCLC cell activity.** The described primary or immortalized NSCLC cells, stably expressing the applied HBO1 shRNA or scramble control shRNAs (“sh-scr”), were established; Expression of *HBO1* mRNA (**A** and **G**) and protein (**B**) was shown. Cells were further maintained in culturing medium for described hours, cell viability (CCK-8 OD, **C** and **H**), colony formation (**D**), proliferation (by examining the EdU-positive nuclei ratio, **E** and **I**) and *in vitro* cell migration (“Transwell” assays, **F** and **J**) were examined. “Pare” stands for parental control cells. *P < 0.05 versus “sh-scr” group. Scale bar = 100 μm (**E** and **F**).

In order to silence HBO1, lentiviral particles encoding six different shRNAs against *HBO1* were individually added to pNSCLC-1 cells. Stable cells were formed after puromycin selection. Testing *HBO1* mRNA expression in the stable cells, by qRT-PCR, showed that three out of the six tested HBO1 shRNAs (sh-HBO1-Sq1/3/5) led to over 90% silencing of *HBO1* mRNA (***P*** < 0.05 versus cells with scramble control shRNA/sh-src, Figure **2A**). HBO1 protein levels were robustly downregulated in sh-HBO1-expressing pNSCLC-1 cells (Figure **2B**). CCK-8 viability was significantly decreased after HBO1 silencing (Figure **2C**). Moreover, pNSCLC-1 cell colony formation was hindered after HBO1 shRNA (Figure **2D**).

In HBO1 shRNA-expressing stable pNSCLC-1 cells, the EdU-positive nuclei ratio was significantly decreased (Figure **2E**), indicating that HBO1 silencing inhibited pNSCLC-1 cell proliferation. HBO1 shRNA potently suppressed pNSCLC-1 cell migration (Figure **2F**). In contrast, the control shRNA (sh-src) failed to significantly alter HBO1 expression (Figure **2A** and **B**) and functions of pNSCLC-1 cells (Figure **2C**-**F**).

To the primary (pNSCLC-2 and pNSCLC-3) and immortalized lines (A549 and H460), sh-HBO1-Sq1 lentiviral particles were added. Stable NSCLC cells were formed after selection. Figure **2G** confirmed that sh-HBO1-Sq1 led to robust *HBO1* mRNA downregulation in the NSCLC cells and it significantly decreased viability (Figure **2H**). Moreover, the ratio of the EdU-positive nuclei was decreased in NSCLC cells bearing the HBO1 shRNA (Figure **2I**). Figure **2J** further showed that sh-HBO1-Sq1 decreased *in vitro* migration of the primary and immortalized NSCLC cells.

### HBO1 silencing provokes NSCLC cell apoptosis

**Figure 3:**
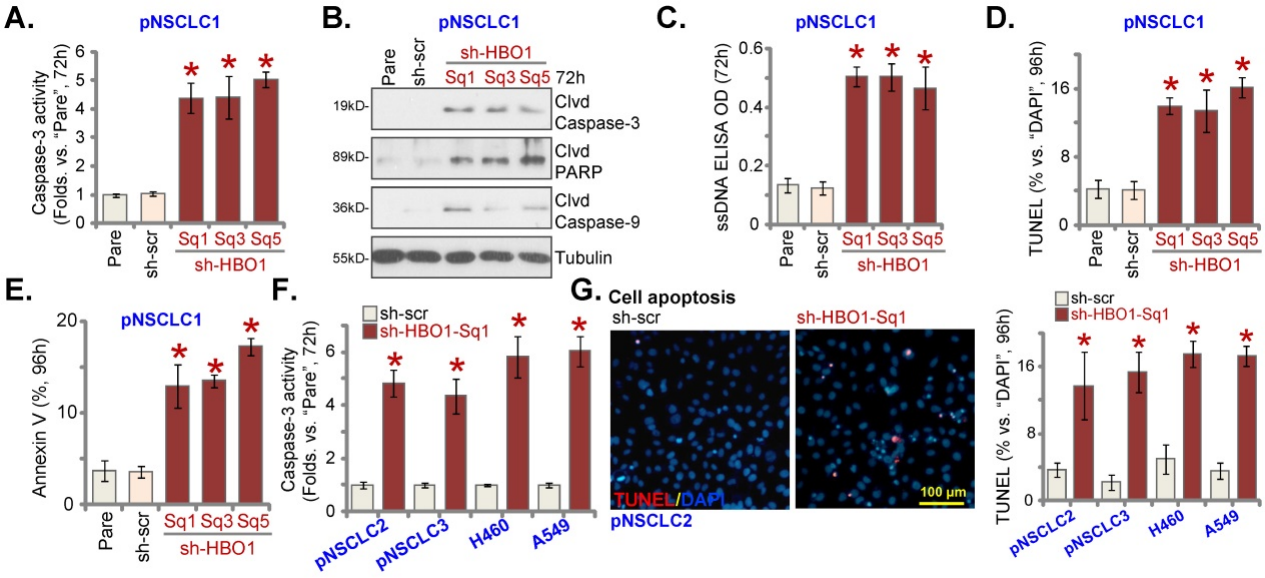
** HBO1 silencing provokes NSCLC cell apoptosis.** The described primary or immortalized NSCLC cells, stably expressing the applied HBO1 shRNA or scramble control shRNAs (“sh-scr”), were established. Cells were further maintained in culturing medium for described hours, the relative caspase-3 activity was tested (**A** and **F**); Expression of listed proteins was tested by Western blotting assays (**B**); Single strand DNA (ssDNA) contents were tested by ELISA assays (**C**). Cell apoptosis was tested by measuring nuclear TUNEL ratio (**D** and **G**) and Annexin V percentage (**E**). “Pare” stands for parental control cells. *P < 0.05 versus “sh-scr” group. Scale bar = 100 μm (**G**).

An early RNA-seq analyses in HBO1-depleted cells have identified over 250 differentially regulated genes, including many anti-apoptosis genes 27. CRISPR/Cas9-induced HBO1 KO induced apoptosis activation in cancer cells 28. In AML cells, depletion of HBO1 caused apoptosis activation 29. The relative caspase-3 activity, Figure **3A**, was significantly increased in pNSCLC-1 cells stably expressing different HBO1 shRNAs (sh-HBO1-Sq1/3/5). In addition, increased cleavages of caspase-3, caspase-9 and PARP were detected in HBO1-silenced pNSCLC-1 cells (Figure **3B**). The single stranded DNA (ssDNA) contents were increased in pNSCLC-1 cells with HBO1 shRNAs (Figure **3C**), indicating increased DNA breakage.

In HBO1 shRNA-expressing stable pNSCLC-1 cells, TUNEL-positive nuclei percentage (% versus DAPI) was significantly increased (Figure **3D**). Figure **3E** demonstrated that HBO1 silencing by targeted shRNAs led to increased Annexin V positive staining, further supporting apoptosis activation. The scramble control shRNA, sh-scr, failed to induce apoptosis activation in pNSCLC-1 cells (Figure **3A**-**E**).

In pNSCLC-2 and pNSCLC-3 primary cells as well as in A549 and H460 immortalized cells, sh-HBO1-Sq1-induced HBO1 silencing increased caspase-3 activity (Figure **3F**). TUNEL-positive nuclei increasing supported apoptosis activation in HBO1-silenced NSCLC cells (Figure **3G**). Taken together, HBO1 shRNA provoked apoptosis activation in human NSCLC cells.

### HBO1 knockout by CRISPR/Cas9 produces significant anti-NSCLC cell activity

**Figure 4:**
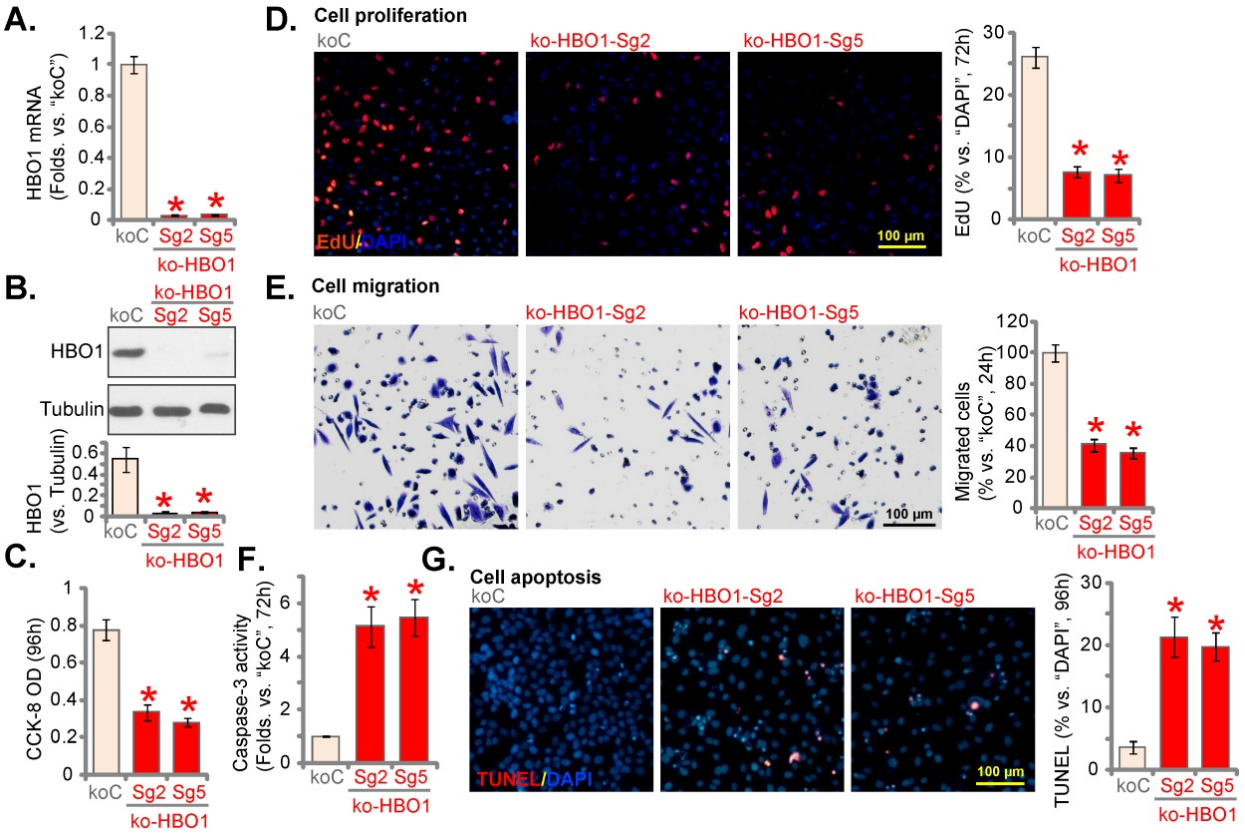
** HBO1 knockout by CRISPR/Cas9 produces significant anti-NSCLC cell activity.** pNSCLC-1 primary cells with the applied lenti-Cas9-HBO1-KO construct (containing ko-HBO1-Sg2 and ko-HBO1-Sg5) or empty vector (“koC”) were established. Expression of *HBO1* mRNA (**A**) and listed proteins (**B**) tested. Cells were further maintained in culturing medium for described hours, CCK-8 OD (**C**), cell proliferation (by testing nuclear EdU staining assays, **D**) and *in vitro* cell migration (**E**) were tested. The relative caspase-3 activity (**F**) and cell apoptosis (by recording TUNEL-positive nuclei ratio, **G**) were examined as well. *P < 0.05 versus “koC” cells. Scale bar = 100 μm (**D**, **E** and **G**).

To completely knockout (KO) HBO1, a set of five different lenti-Cas9-HBO1-KO constructs, encoding different sgRNAs targeting *HBO1*, were individually transduced to pNSCLC-1 cells. Single stable cells were formed. Among the five tested sgRNAs, HBO1 sgRNA-2 (“Sg2”) and HBO1 sgRNA-5 (“Sg5”) resulted in complete *HBO1* mRNA depletion (Figure **4A**). HBO1 protein levels were almost depleted (Figure **4B**). HBO1 KO decreased CCK-8 viability (Figure **4C**) and robustly inhibited cell proliferation (EdU-positive nuclei percentage decreasing, Figure **4D**) in pNSCLC-1 cells. HBO1 KO potently inhibited pNSCLC-1 cell* in vitro* cell migration (Figure **4E**). HBO1 KO induced caspase-3 activation (Figure **4F**) and apoptosis (Figure **4G**) in pNSCLC-1 cells.

### Ectopic HBO1 overexpression further promotes NSCLC cell proliferation and migration

**Figure 5:**
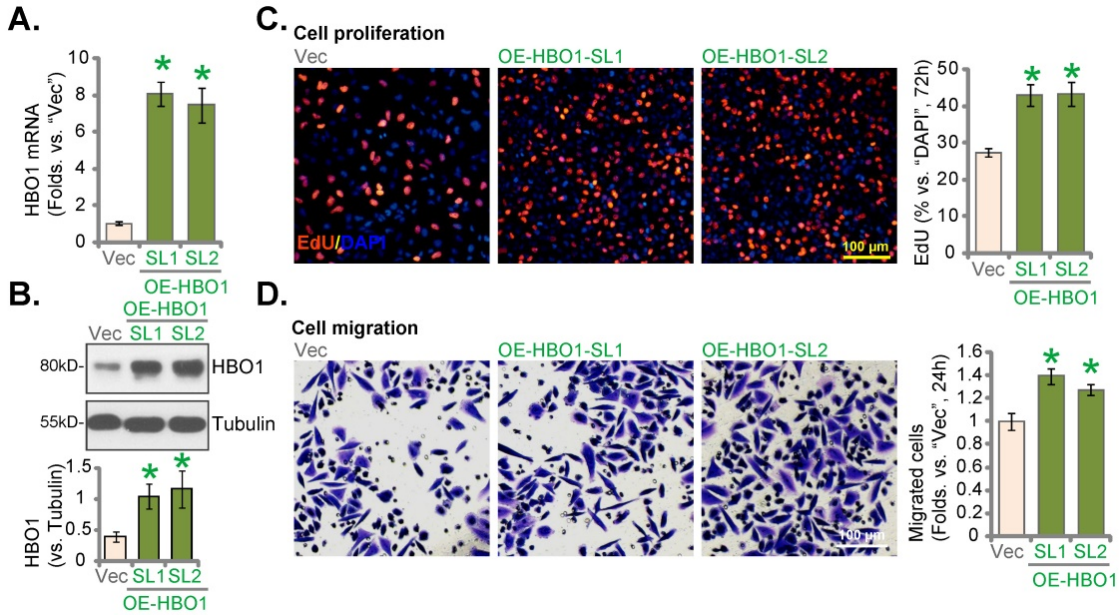
** Ectopic HBO1 overexpression further promotes NSCLC cell proliferation and migration.** pNSCLC-1 primary cells stably expressing the lentiviral HBO1-expressing construct (“OE-HBO1-SL1” and “OE-HBO1-SL2”, two selections) or the empty vector (“Vec”), were established, with expression of *HBO1* mRNA (**A**) and protein (**B**) tested. Cells were further maintained in culturing medium for described hours, cell proliferation (EdU assays, **C**) and *in vitro* cell migration (**D**) were tested. *P < 0.05 versus “Vec” cells. Scale bar = 100 μm (**C** and **D**).

Next a lentiviral HBO1-expressing construct (from Dr. Cao 24) was transduced to pNSCLC-1 cells. Two stable cell selections, OE-HBO1-SL1 and OE-HBO1-SL2, were formed after selection. As shown *HBO1* mRNA increased over seven fold in OE-HBO1 pNSCLC-1 cells (Figure **5A**). HBO1 protein overexpression was detected as well (Figure **5B**). HBO1 overexpression increased cell proliferation (evidenced by the EdU-positive nuclei percentage increasing, Figure **5C**) and *in vitro* migration (Figure **5D**) in pNSCLC-1 cells.

### HBO1 is important for H3-H4 acetylation and expression of several oncogenic genes in NSCLC cells

**Figure 6:**
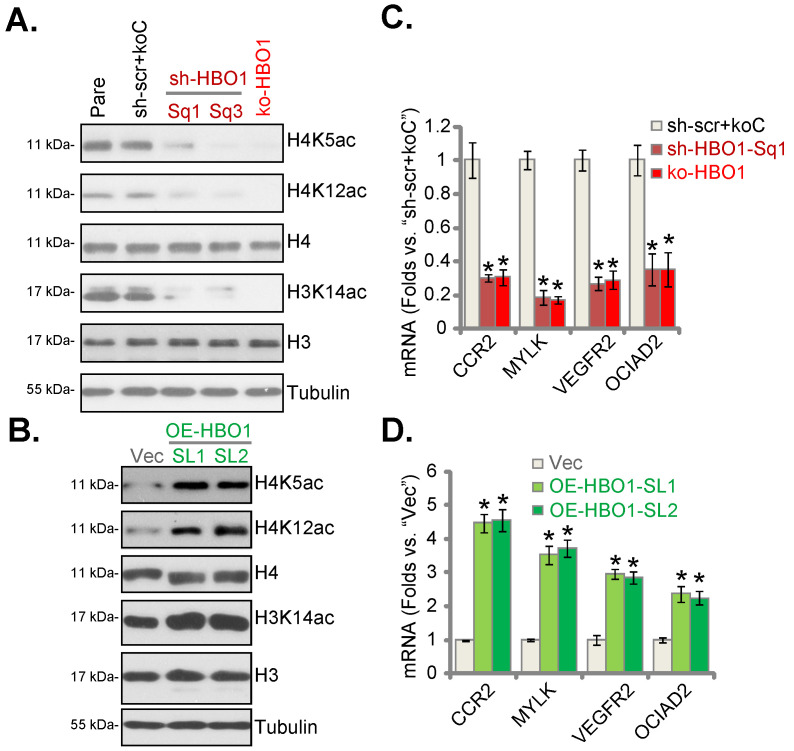
** HBO1 is important for H3-H4 acetylation and expression of several oncogenic genes in NSCLC cells.** The stable pNSCLC-1 cells with the described HBO1 shRNA (sh-HBO1-Sq1 or sh-HBO1-Sq3), the described lenti-Cas9-HBO1-KO construct (ko-HBO1-Sg2) or the scramble control shRNA plus empty vector (“sh-scr+koC”) were established. Alternatively, pNSCLC-1 cells with the HBO1-expressing lentiviral construct (OE-HBO1-SL1 and OE-HBO1-SL2, two cell selection) or the empty vector (“Vec”) were established as well; Expression of the listed proteins and mRNAs in the described cells were tested by Western blotting (**A** and **B**) and qRT-PCR (**C** and **D**) assays, respectively. *P < 0.05 versus “sh-scr+koC”/“Vec” cells.

HBO1 catalyzes H4 acetylation (H4K5ac and H4K12ac) and H3K14ac in human cancer 22, 24, 28. As shown HBO1 silencing or CRISPR/Cas9-mediated HBO1 KO largely inhibited H4K5ac, H4K12ac and H3K14ac in pNSCLC-1 cells (Figure **6A**). Conversely, their levels were increased in HBO1-overexpressed pNSCLC-1 cells (Figure **6B**). Total H3 and H4 expression was however not significantly altered (Figure **6A** and **B**).

RNA-Seq studies have tested differentially-regulated mRNAs in control cells versus HBO1-depleted cells 27, 28. Many of these HBO1-dependent genes could exert oncogenic/cancer-promoting functions or being upregulated in NSCLC, including *C‑C chemokine receptor type 2* (*CCR2*) 30, 31, *myosin light chain kinase* (*MYLK*) 32, *VEGFR2* 33, 34, *ovarian cancer immunoreactive antigen domain containing 2* (*OCIAD2*) 35*.* Here, mRNA levels of *CCR2*, *MYLK*, *VEGFR2* and *OCIAD2* were dramatically reduced in HBO1-silenced and HBO1-KO pNSCLC-1 cells (Figure **6C**). In contrast, their expression was robustly elevated in OE-HBO1-SL1 and OE-HBO1-SL2 cells (Figure **6D**). These results suggest that HBO1 is vital for H3-H4 acetylation and expression of multiple oncogenic genes in NSCLC cells.

### HBO1 silencing inhibits NSCLC xenograft growth in mice

A549 cells (at six million cells of each mouse) were inoculated to the flanks of the nude mice. Within 21 days A549 xenografts were established (tumor volume at close to100 mm^3^). The xenograft-bearing mice received intratumoral injection of AAV-packed HBO1 shRNA (“aav-shHBO1-Sq1”) or AAV scramble control shRNA (“aav-sh-scr”), with 10 mice in each group. Virus injection was carried out daily for six consecutive days (“Day-0” to “Day-5”).

**Figure 7:**
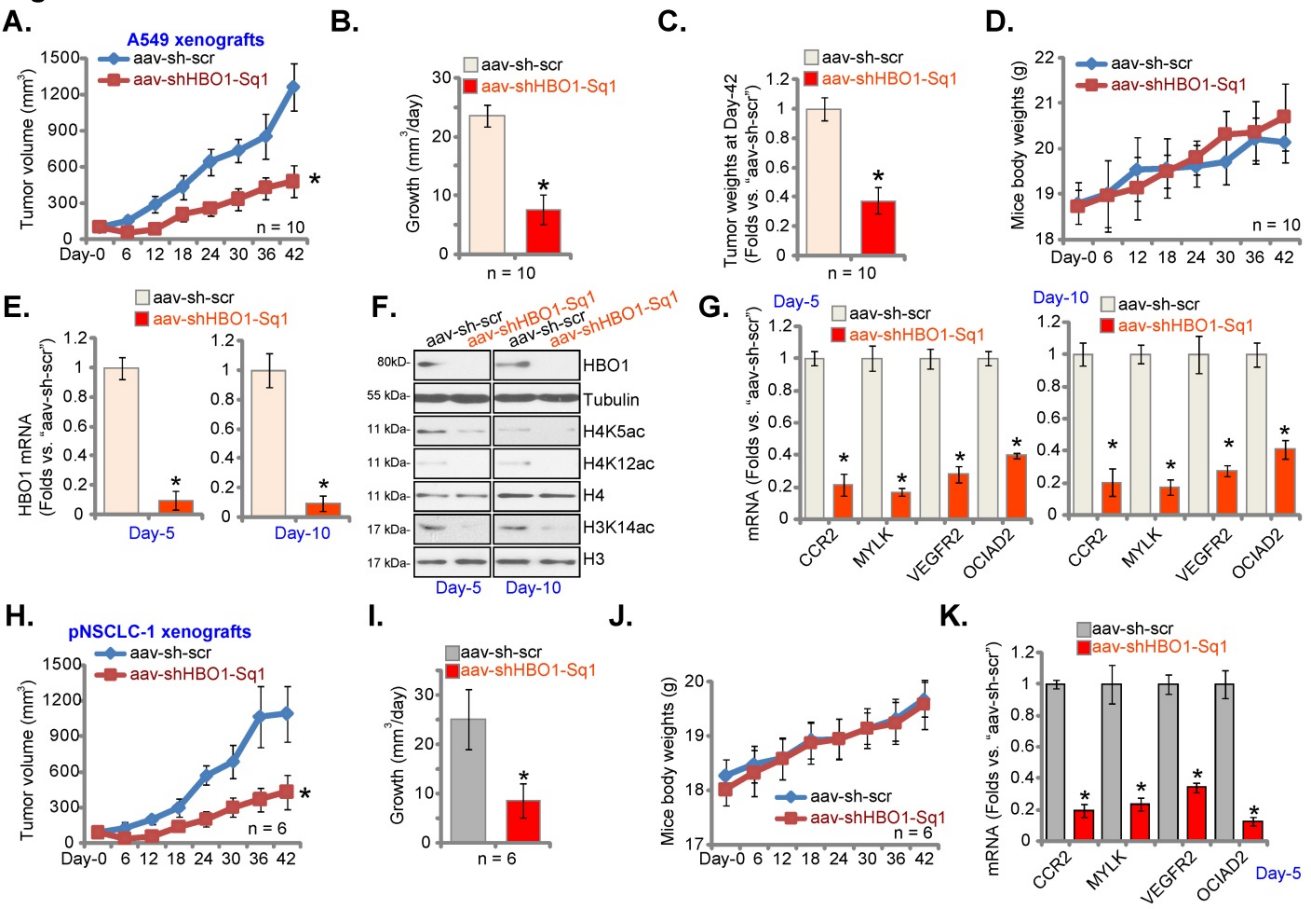
** HBO1 silencing inhibits NSCLC xenograft growth in mice.** A549 xenograft-bearing nude mice (**A**-**G**) or the pNSCLC-1 xenograft-bearing nude mice (**H**-**K**) were subjected to intratumoral injection of AAV-packed HBO1 shRNA (“aav-shHBO1-Sq1”) or AAV-packed scramble control shRNA (“aav-sh-scr”). Virus injection was performed daily for six days. Tumor volumes (**A** and **H**) and mice body weights (**D** and **J**) were recorded every six days (total of 42 days). The estimated daily tumor growth was calculated (**B** and **I**). At Day-42, A549 tumors were isolated and individually weighted (**C**). The described tumors were isolated, and tumor lysates were obtained. Expression of the listed mRNAs (**E**, **G** and **K**) and proteins (**F**) was tested. *P < 0.05 versus “aav-sh-scr” group.

Figure **7A**, demonstrated that injection of HBO1 shRNA AAV largely inhibited A549 xenograft growth. The estimated daily tumor growth was calculated as described 25. Figure **7B** confirmed that HBO1 shRNA potently suppressed A549 xenograft growth. At Day-42, A549 tumors were isolated. Tumors in aav-shHBO1-Sq1 group mice were lighter than those in aav-sh-scr group mice (Figure **7C**). Figure **7D** confirmed that the animal body weights were not different significantly among the two groups.

At “Day-5” and “Day-10”, one tumor per group was carefully isolated 6h after virus injection, and fresh tumor lysates were achieved. *HBO1* mRNA and protein levels (Figure **7E-F**) were significantly decreased in aav-shHBO1-Sq1-injected xenograft tissues, where H4K5ac, H4K12ac and H3K14ac levels were largely inhibited (Figure **7F**). H3 and H4 protein levels were however unchanged (Figure **7F**). In addition, mRNA levels of HBO1-dependent genes, *CCR2*, *MYLK*, *VEGFR2* and *OCIAD2*, were robustly reduced in HBO1-shRNA-injected A549 xenografts (Figure **7G**). Thus, HBO1 silencing inhibited H3-H4 acetylation and decreased expression of HBO1-dependent genes in A549 xenografts.

Next, pNSCLC-1 primary cells were *s.c.* injected to the nude mice (again at six million cells of each mouse). Three weeks after cell implantation, pNSCLC-1 xenografts were formed and patient-derived xenograft model was established. Mice were thereafter subject to the intratumoral injection of aav-shHBO1-Sq1 or aav-sh-scr. Virus injection was carried out daily for six consecutive days (“Day-0” to “Day-5”). Recording weekly tumor volumes, Figure **7H**, demonstrated that injection of the HBO1 shRNA virus potently inhibited pNSCLC-1 xenograft growth in the experimental animals. The estimated daily tumor growth was inhibited following HBO1 shRNA (Figure **7I**). The body weights of the nude mice were again indifferent (Figure **7J**). At experimental Day-5, six hours after virus injection, one tumor of each group was isolated. *CCR2*, *MYLK*, *VEGFR2* and *OCIAD2* mRNA levels were reduced in HBO1 shRNA virus-injected pNSCLC-1 xenografts (Figure **7K**).

### WM-3835 induces significant anti-cancer activity in NSCLC cells

**Figure 8:**
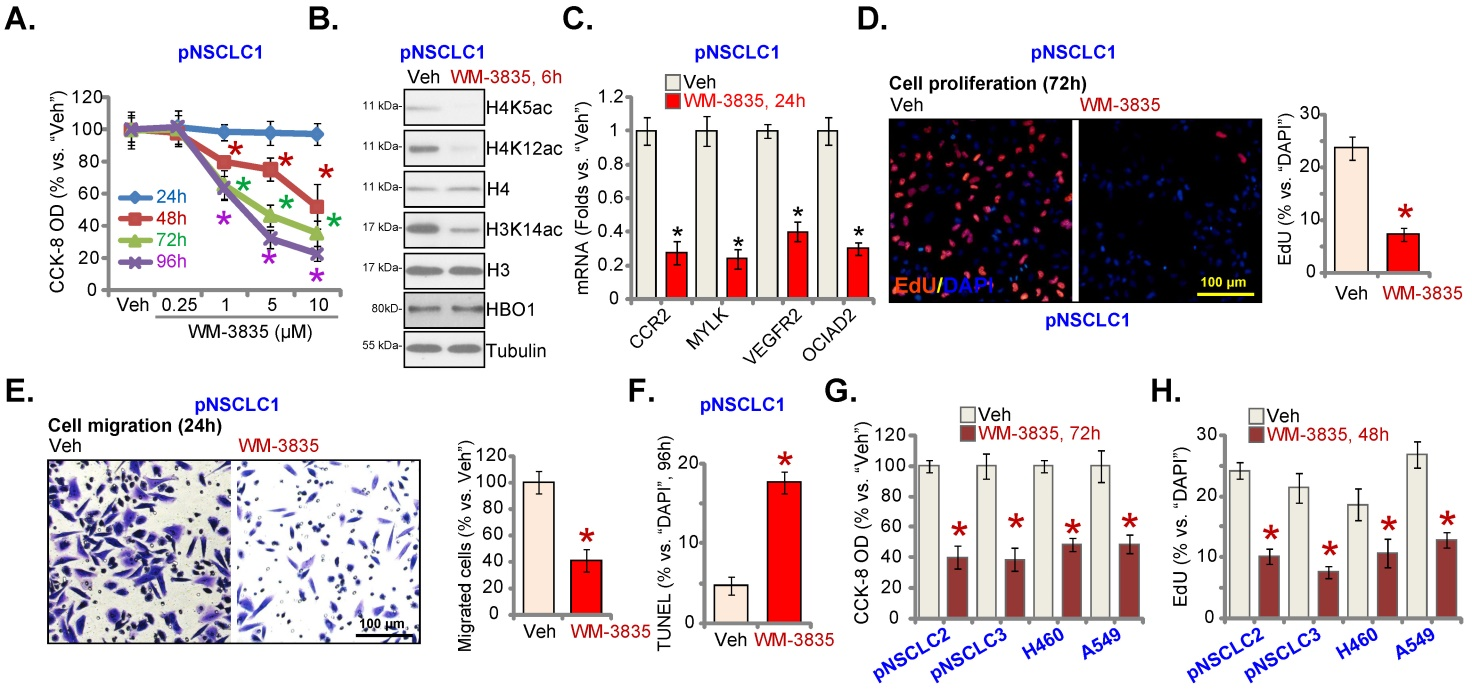
** WM-3835 induces significant anti-cancer activity in NSCLC cells.** The listed primary or immortalized NSCLC cells were treated with WM-3835 (at 5 μM, expect for **A**) or the vehicle control (“Veh”, 0.5% DMSO), and maintained in culturing medium for described hours; Cell viability was tested by CCK-8 assays (**A** and **G**), and expression of listed proteins and mRNAs were shown (**B** and **C**); Cell proliferation (**D** and **H**),*in vitro* cell migration (**E**) and apoptosis (**F**, by measuring TUNEL-positive nuclei percentage) were tested by the described assays. “Pare” stands for parental control cells. * ***P*** < 0.05 versus “Veh” group. Scale bar = 100 μm (**D** and **E**).

At last, the effect of a first-in-class small molecule inhibitor of HBO1 24, 28 WM-3835 was studied. Figure **8A** demonstrated that WM-3835 decreased pNSCLC1 CCK-8 viability in a time-dependent manner. It required 48-96h for the HBO1 inhibitor to exert a significant effect in decreasing cell viability (Figure **8A**). Moreover, WM-3835-induced cell viability reduction was concentration dependent (Figure **8A**). The IC-50 was close to 5 μM. H3 and H4 histone acetylation was largely inhibited by WM-3835 (Figure **8B**). HBO1 protein expression was unchanged (Figure **8B**). *CCR2*, *MYLK*, *VEGFR2* and *OCIAD2* mRNA levels were dramatically decreased in WM-3835-treated pNSCLC1 cells (Figure **8C**).

Furthermore, treatment with the HBO1 inhibitor inhibited pNSCLC1 cell proliferation and *in vitro* cell migration, examined by the nuclear EdU-staining (Figure **8D**) and “Transwell” (Figure **8E**) assays, respectively. WM-3835 increased the percentage of the TUNEL-positive nuclei in pNSCLC1 cells, suggesting apoptosis activation (Figure **8F**). In pNSCLC-2 and pNSCLC-3 primary cells as well as in the immortalized cells (A549 and H460), WM-3835 (5 μM) resulted in significant viability reduction (Figure **8G**) and proliferation arrest (EdU-positive cell decreasing, Figure **8H**). Thus, the HBO1 inhibitor WM-3835 induced significant anti-cancer activity in different human NSCLC cells.

## Discussion

By catalyzing histone lysine acetylation, HBO1 and other HATs are essential in transcriptional regulation of gene expression and various key biological processes 36-38. Dysregulation of HATs is commonly detected in NSCLC. One HAT hMOF is overexpressed in NSCLC, and it is essential for growth and tumorigenesis of NSCLC 39, 40. CPTH6, a HAT inhibitor, preferentially inhibited lung cancer stem-like cell growth and viability 41. Another HAT inhibitor, A485, enhanced the sensitivity of TRAIL in NSCLC cells 42. C646, a p300 HAT inhibitor, radio-sensitized NSCLC cells by facilitating mitotic catastrophe 43.

HBO1 is vital for H3 and H4 acetylation, gene transcription, DNA replication and repair 15, 44. Through directly binding to ORC1, HBO1 interacts with pre-replication complex proteins, including MCM2 19 and chromatin licensing and DNA replication Factor 1 45, thereby promoting DNA replication. HBO1 acetylated ORC2, MCM2, CDC6, and geminin, and it was essential for replication licensing 46. By acting as the positive regulator of centromeric Centromere Protein A, HBO1 inhibited inactivation of SUV39H1-mediated centromere 47. In addition, ATR-dependent HBO1 phosphorylation is import for DNA repair 48.

Recent studies implied a pivotal role of HBO1 in tumorigenesis 15. MacPherson *et al.,* showed that HBO1 and several known members of the HBO1 protein complex served as the critical regulators for leukaemia stem cells 28. In acute myeloid leukemia (AML) cells, HBO1 depletion led to a rapid and complete loss of H3K14ac and H4K12ac, and caused proliferation inhibition, apoptosis and cell differentiation 29. HBO1 was required for Wnt/beta-catenin signaling activation and proliferation of bladder cancer cells 49. HBO1 expression was elevated in HCC and it was important for cancer cell growth 22. Gao* et al.,* have proposed HBO1 as a novel oncogenic gene for osteosarcoma. HBO1 overexpression in osteosarcoma was required for cell growth *in vitro* and *in vivo* 24.

Our results support that HBO1 could be a key therapeutic target of NSCLC. TCGA and Oncomine bioinformatics analyses have shown that *HBO1* is overexpressed in human NSCLC tissues. Furthermore, in local NSCLC tumor tissues HBO1 is significantly overexpressed. HBO1 overexpression was detected in different established and immortalized human NSCLC cells. In different NSCLC cells, HBO1 shRNA or KO inhibited cell viability, proliferation and migration, and provoked apoptosis activation. Conversely, HBO1 overexpression further increased NSCLC cell proliferation and migration. Importantly, AAV-packed HBO1 shRNA intratumoral injection largely hindered NSCLC xenograft tumor growth in nude mice. WM-3835, a first-in-class small molecule HBO1 inhibitor, potently inhibited NSCLC cell growth.

A large number of HBO1-dependent genes, *CCR2*, *MYLK*, *VEGFR2* and *OCIAD2*, could exert tumor-promoting activity in NSCLC 15, 22, 28, 44, 50. VEGFR2 is a well-established oncogene in NSCLC 33, 34. Moreover, CCR2 expression was shown to be upregulated in NSCLC tissues and cells 31, 51. CAS445479-97-0, a CCR2 antagonist, potently inhibited viability, motility and invasion of NSCLC cells 31. Conversely, the CCR2 ligand CCL2 promoted NSCLC cell proliferation, migration and invasion by promoting MMP-9 expression 31. Tan *et al.,* showed that expression of MYLK, a HBO1-dependent gene, in stages III and IV NSCLC was significantly higher than that in stages I and II NSCLC 32. Furthermore, MYLK expression in NSCLC with lymphatic metastasis was higher 32. Moreover, OCIAD2 was associated with progression of early-stage lung adenocarcinoma and overexpression predicted poorer prognosis in patients 35, 52. Here we showed that *CCR2*, *MYLK*, *VEGFR2* and *OCIAD2* expression was significantly decreased in NSCLC cells with HBO1 silencing or KO. They were however increased after HBO1 overexpression. Moreover, the expression of these genes was significantly reduced in HBO1-shRNA AAV-injected NSCLC xenograft tissues. Therefore, HBO1-driven NSCLC cell growth could be due to its role in promoting H3-H4 acetylation and expression of important NSCLC-promoting genes.

## Conclusion

Overexpressed HBO1 induces histone acetylation and is important for NSCLC cell growth.

## Supplementary Material

Supplementary figure.
