# Supplementary figures and images for: HBO1 induces histone acetylation and is important for non-small cell lung cancer cell growth
Source: Int J Biol Sci. 2022 May 9;18(8):3313–23. doi: 10.7150/ijbs.72526 (PMC9134900; doi:10.7150/ijbs.72526)

# Figure S1

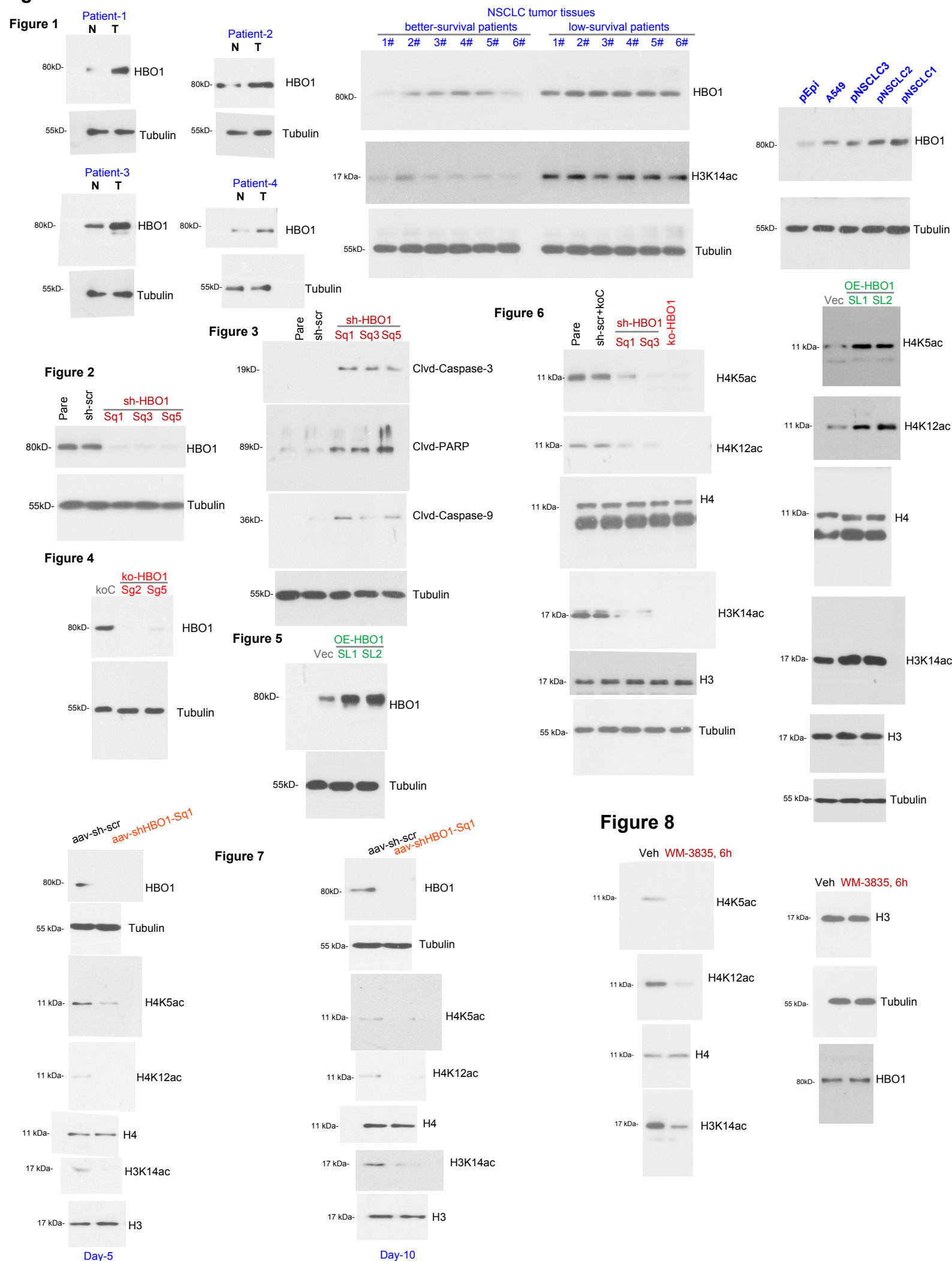

**Figure S1: The uncropped blotting images of the study.**

Supplement: Supplementary file 1 — Supplementary figure. [file ijbsv18p3313s1.pdf]
